# Supplementary material for: Waning of SARS-CoV-2 booster viral-load reduction effectiveness
Source: Nat Commun. 2022 Mar 4;13:1237. doi: 10.1038/s41467-022-28936-y (PMC8897467; doi:10.1038/s41467-022-28936-y)
Supplement: Supplementary file 1 — Supplementary Information [file 41467_2022_28936_MOESM1_ESM.pdf]

Supplementary Information for

"Waning of SARS-CoV-2 booster viral-load reduction effectiveness"

Matan Levine-Tiefenbrun[1,2], Idan Yelin[1], Hillel Alapi[3], Esma Herzel[3], Jacob Kuint[2,3], Gabriel Chodick[2,3], Sivan Gazit[3], Tal Patalon*[3], Roy Kishony*[1,4]

[1] Faculty of Biology, Technion - Israel Institute of Technology, Haifa, Israel

[2] Sackler Faculty of Medicine, Tel-Aviv University, Tel-Aviv, Israel

[3] Maccabitech, Maccabi Health Services, Tel Aviv, Israel

[4] Faculty of Computer Science, Technion - Israel Institute of Technology, Haifa, Israel

* corresponding authors. Email: patalon_t@mac.org.il, rkishony@technion.ac.il.

This document includes:

Supplementary Figures 1 to 3

Supplementary Table 1

Supplementary Figure 1. Association of infection Ct with two-dose vaccination and with the booster, for the *N* and *E* genes. Ct regression coefficients of the *N* gene (a) and the *E* gene (b), indicating an infection Ct relative to unvaccinated control group (dashed line). Coefficients were obtained by multivariate linear regression analysis, adjusting for age, sex and calendric date (Methods; n = 22,657). Error bars represent one standard error of the mean. All P values are two sided: 'ns', not significant, * P < 0.05, ** P < 0.01, *** P < 0.001.

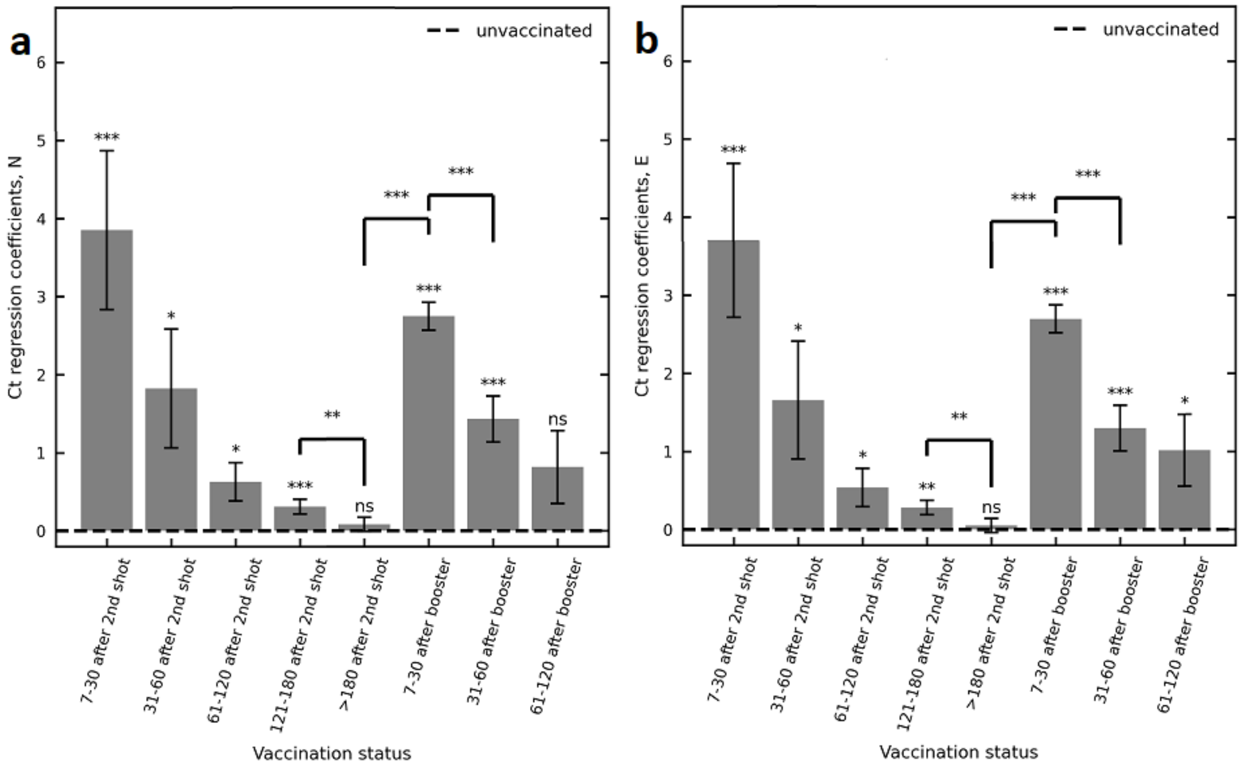

Supplementary Figure 2. Age distributions of individuals infected in the 3 post-booster time bins were similar. Shown are the age distributions of individuals infected 7-30 days (blue), 31-60 days (orange) and 61-120 days (green) post-booster.

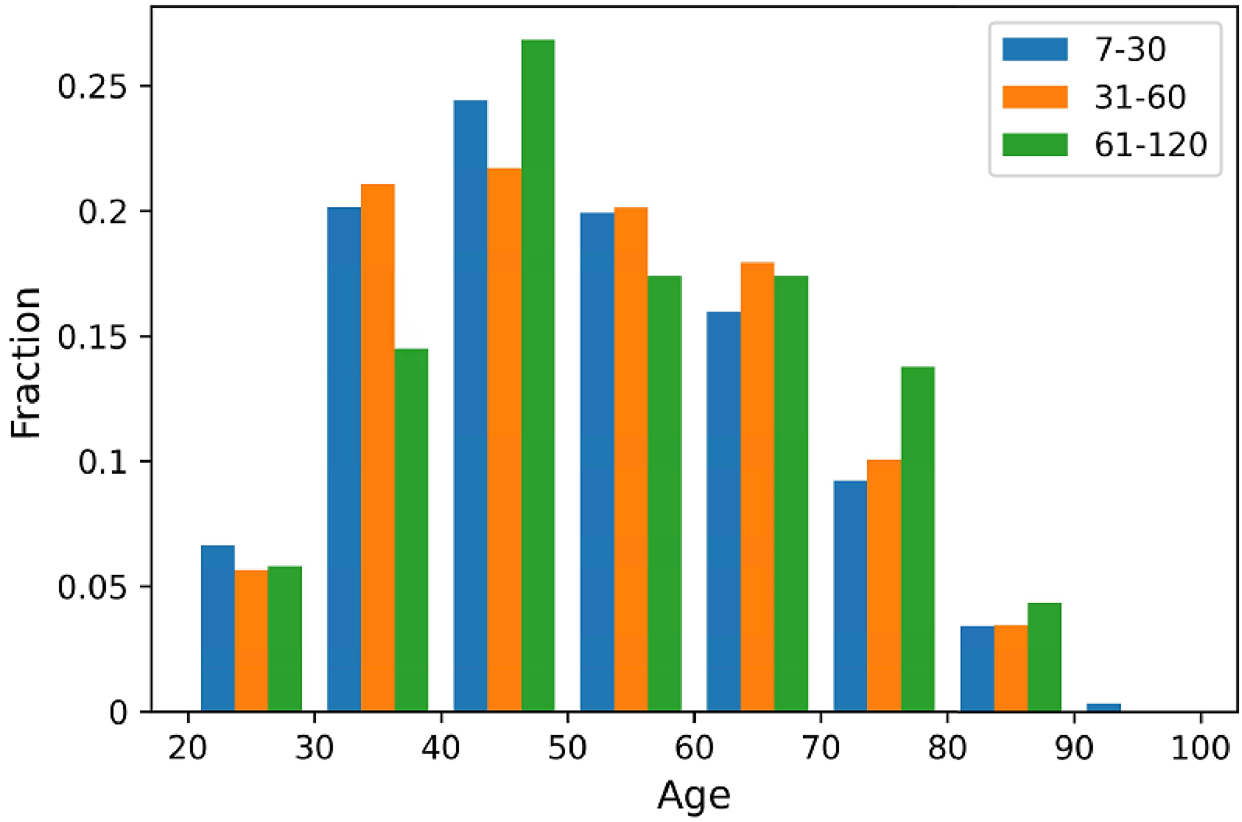

Supplementary Figure 3. Regression coefficients for the association of Ct with two-dose vaccination and with the booster for non-hospitalized patients. Same model as in Fig. 1 and Supplementary Figure 1, but excluding patients hospitalized due to COVID-19 (n = 22,271). Ct regression coefficients are shown for the RdRp (a), N (b) and E (c) genes. Error bars represent one standard error of the mean. All P values are two sided: ‘ns’, not significant, * P < 0.05, ** P < 0.01, *** P < 0.001.

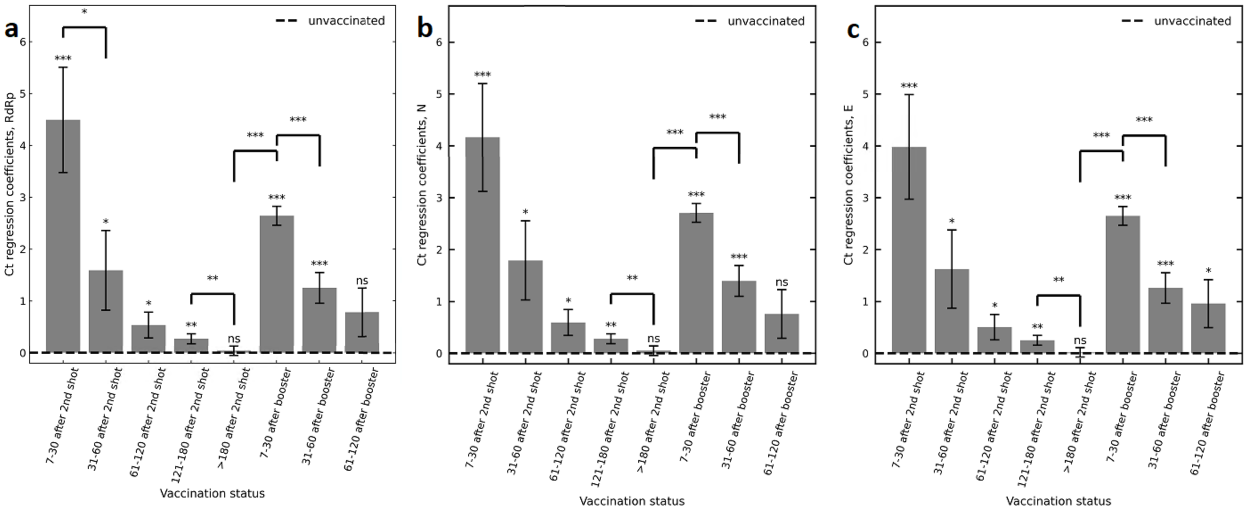

Supplementary Table 1: Study population.

	2-dose-vaccinated					Booster-Vaccinated			Unvaccinated
	**7-30** post 2nd shot	**31-60** post 2nd shot	**61-120** post 2nd shot	**121-180** post 2nd shot	**>180** post 2nd shot	**7-30** post booster shot	**31-60** post booster shot	**61-120** post booster shot	
**# (100%)**	25	43	456	8,076	7,438	934	318	138	5,229
**# of male (%)**	8 (32)	18 (42)	154 (34)	3,462 (43)	3,321 (45)	477 (51)	149 (47)	68 (49)	2,189 (42)
**# of female (%)**	17 (68)	25 (58)	302 (66)	4,614 (57)	4,117 (55)	457 (49)	169 (53)	70 (51)	3,040 (58)
**Age in years (STD)**	43.2 (19.8)	39.8 (11.1)	36.8 (13.8)	39.0 (13.2)	43.6 (14.7)	50.6 (15.4)	50.9 (15.3)	52.9 (15.4)	40.5 (14.1)
**Mean RdRp Ct (STD)**	30.8 (4.5)	28.4 (5.0)	27.2 (4.8)	26.9 (5.0)	26.8 (5.0)	29.4 (4.7)	28.5 (4.4)	28.9 (4.5)	26.8 (5.0)
**Mean N Ct (STD)**	29.0 (4.1)	27.1 (4.8)	25.9 (4.9)	25.5 (5.0)	25.3 (5.0)	27.9 (4.7)	27.1 (4.5)	27.4 (4.5)	25.3 (5.0)
**Mean E Ct (STD)**	26.4 (4.2)	24.6 (4.9)	23.3 (4.8)	23.0 (4.9)	22.9 (5.0)	25.5 (4.6)	24.6 (4.5)	25.1 (4.4)	22.9 (5.0)
