# Supplementary material for: Waning of SARS-CoV-2 booster viral-load reduction effectiveness
Source: Nat Commun. 2022 Mar 4;13:1237. doi: 10.1038/s41467-022-28936-y (PMC8897467; doi:10.1038/s41467-022-28936-y)
Supplement: Supplementary file 3 — Reporting Summary [file 41467_2022_28936_MOESM3_ESM.pdf]

nature portfolio

Corresponding author(s): Roy Kishony, Tal Patalon

Last updated by author(s): Feb 14, 2022

Reporting Summary

Nature Portfolio wishes to improve the reproducibility of the work that we publish. This form provides structure for consistency and transparency in reporting. For further information on Nature Portfolio policies, see our Editorial Policies and the Editorial Policy Checklist.

Statistics

For all statistical analyses, confirm that the following items are present in the figure legend, table legend, main text, or Methods section.

n/a	Confirmed	
☐	☒	The exact sample size (*n*) for each experimental group/condition, given as a discrete number and unit of measurement
☒	☐	A statement on whether measurements were taken from distinct samples or whether the same sample was measured repeatedly
☐	☒	The statistical test(s) used AND whether they are one- or two-sided<br>*Only common tests should be described solely by name; describe more complex techniques in the Methods section.*
☐	☒	A description of all covariates tested
☐	☒	A description of any assumptions or corrections, such as tests of normality and adjustment for multiple comparisons
☐	☒	A full description of the statistical parameters including central tendency (e.g. means) or other basic estimates (e.g. regression coefficient) AND variation (e.g. standard deviation) or associated estimates of uncertainty (e.g. confidence intervals)
☐	☒	For null hypothesis testing, the test statistic (e.g. *F*, *t*, *r*) with confidence intervals, effect sizes, degrees of freedom and *P* value noted<br>*Give P values as exact values whenever suitable.*
☒	☐	For Bayesian analysis, information on the choice of priors and Markov chain Monte Carlo settings
☒	☐	For hierarchical and complex designs, identification of the appropriate level for tests and full reporting of outcomes
☒	☐	Estimates of effect sizes (e.g. Cohen's *d*, Pearson's *r*), indicating how they were calculated

Our web collection on statistics for biologists contains articles on many of the points above.

Software and code

Policy information about availability of computer code

Data collection	SQL Server Managment Studio v18.5.1, Seegene proprietary software: CFX Manager Software V3.1.
Data analysis	Python 3.7.1. Including: scipy (v1.1.0) and statsmodels (v0.9.0).<br>Code for analysis can be found at: https://github.com/matanlevine/Waning-of-SARS-CoV-2-booster-viral-load-reduction-effectiveness

For manuscripts utilizing custom algorithms or software that are central to the research but not yet described in published literature, software must be made available to editors and reviewers. We strongly encourage code deposition in a community repository (e.g. GitHub). See the Nature Portfolio guidelines for submitting code & software for further information.

Data

Policy information about availability of data

All manuscripts must include a data availability statement. This statement should provide the following information, where applicable:

- Accession codes, unique identifiers, or web links for publicly available datasets
- A description of any restrictions on data availability
- For clinical datasets or third party data, please ensure that the statement adheres to our policy

According to Israel Ministry of Health regulations, individual-level data cannot be shared openly. Specific requests for remote access to de-identified data should be referred to KSM, the Maccabi Healthcare Services Research and Innovation Center. Requests sent to Sarit Chehanowitz (chehanow_s@mac.org.il) will be considered within 21 d pending IRB approval and Maccabi Healthcare Services regulations. Source data are provided with this paper.

March 2021

Field-specific reporting

Please select the one below that is the best fit for your research. If you are not sure, read the appropriate sections before making your selection.

☒ Life sciences ☐ Behavioural & social sciences ☐ Ecological, evolutionary & environmental sciences

For a reference copy of the document with all sections, see nature.com/documents/nr-reporting-summary-flat.pdf

Life sciences study design

All studies must disclose on these points even when the disclosure is negative.

Sample size	Sample size was not predetermined or restricted. It was only limited by the number of patients with breakthrough infections available in the database.
Data exclusions	We have excluded patients under 20 years old, as this group wasn't approved yet for booster shot, and was lately approved for the vaccine at all. This exclusion criterion was pre-established.
Replication	We report the results of an observational study.
Randomization	Not randomized. In our analysis we used linear regression model, making randomization unneeded.
Blinding	We report the results of an observational study.

Reporting for specific materials, systems and methods

We require information from authors about some types of materials, experimental systems and methods used in many studies. Here, indicate whether each material, system or method listed is relevant to your study. If you are not sure if a list item applies to your research, read the appropriate section before selecting a response.

Materials & experimental systems

n/a	Involved in the study	
☒	☐	Antibodies
☒	☐	Eukaryotic cell lines
☒	☐	Palaeontology and archaeology
☒	☐	Animals and other organisms
☐	☒	Human research participants
☒	☐	Clinical data
☒	☐	Dual use research of concern

Methods

n/a	Involved in the study	
☒	☐	ChIP-seq
☒	☐	Flow cytometry
☒	☐	MRI-based neuroimaging

Human research participants

Policy information about studies involving human research participants

Population characteristics	Patients above 20 years old infected with SARS-CoV-2: 5,229 unvaccinated (2,189 males and 3,040 female); 16,038 2-dose vaccinated (6,963 males and 9,075 females); 1,390 booster vaccinated (694 males and 696 females).<br>Ages:<br>20-30: 5,452 patients<br>30-40: 5,901 patients<br>40-50: 5,190 patients<br>50-60: 3,398 patients<br>60-70: 1,607 patients<br>70-80: 820 patients<br>80-90: 250 patients<br>90-100: 39 patients
Recruitment	This is an observational study involving electronic health records analysis.
Ethics oversight	This study was approved by the MHS (Maccabi Health Services) Institutinal Review Board (IRB).

Note that full information on the approval of the study protocol must also be provided in the manuscript.
